# Patient-reported and doctor-reported symptoms when faecal immunochemical tests are requested in primary care in the diagnosis of colorectal cancer and inflammatory bowel disease: a prospective study

**DOI:** 10.1186/s12875-020-01194-x

**Published:** 2020-07-01

**Authors:** Cecilia Högberg, Pontus Karling, Jörgen Rutegård, Mikael Lilja

**Affiliations:** 1 Department of Public Health and Clinical Medicine, Unit of Research, Education and Development – Östersund, Östersund Hospital, Umeå University, Umeå, Sweden; 2 Department of Public Health and Clinical Medicine, Division of Medicine, Umeå University, Umeå, Sweden; 3 Department of Surgical and Perioperative Sciences, Umeå University, Umeå, Sweden

**Keywords:** Colorectal neoplasms, Faecal immunochemical test, Gastrointestinal symptoms, Occult blood, Primary care, Rectal bleeding

## Abstract

**Background:**

Rectal bleeding and a change in bowel habits are considered to be alarm symptoms for colorectal cancer and they are also common symptoms for inflammatory bowel disease. However, most patients with these symptoms do not have any of these diseases. Faecal immunochemical tests (FITs) for haemoglobin are used as triage tests in Sweden and other countries but little is known about the symptoms patients have when FITs are requested.

**Objective:**

Firstly, to determine patients’ symptoms when FITs are used as triage tests in primary care and whether doctors record the symptoms that patients report, and secondly to evaluate the association between symptoms, FIT results and possible prediction of colorectal cancer or inflammatory bowel disease.

**Methods and materials:**

This prospective study included 364 consecutive patients for whom primary care doctors requested a FIT. Questionnaires including gastrointestinal symptoms were completed by patients and doctors.

**Results:**

Concordance between symptoms reported from patients and doctors was low. Rectal bleeding was recorded by 43.5% of patients versus 25.6% of doctors, FITs were negative in 58.3 and 52.7% of these cases respectively. The positive predictive value (PPV) of rectal bleeding recorded by patients for colorectal cancer or inflammatory bowel disease was 9.9% (95% confidence interval [CI] 5.2–14.7); for rectal bleeding combined with a FIT the PPV was 22.6% (95% CI 12.2–33.0) and the negative predictive value (NPV) was 98.9% (95% CI 96.7–100). For patient-recorded change in bowel habits the PPV was 6.1% (95% CI 2.4–9.8); for change in bowel habits combined with a FIT the PPV was 18.2% (95% CI 9.1–30.9) and the NPV 100% (95% CI 90.3–100).

**Conclusions:**

Doctors should be aware that, during consultations, they do not record all symptoms experienced by patients. FITs requested in primary care, when found positive, may potentially be of help in prioritising referrals, also when patients present with rectal bleeding or change in bowel habits.

## Background

Colorectal cancer (CRC) is the third most common cancer worldwide [1]. The cumulative life prevalence is approximately 5% [2]. Early stage CRC is often asymptomatic, and when symptoms develop it is of importance to determine a diagnosis without delay, as longer diagnostic intervals may result in higher mortality [3].

Rectal bleeding, change in bowel habits and weight loss are considered to be alarm symptoms for CRC, and guidelines on suspected CRC recommend that patients who experience these symptoms be referred to secondary care [4–6]. However, these symptoms are common amongst the general public and in patients consulting primary care [7, 8], and the majority of these patients do not have significant colorectal disease [9, 10]. Additionally, many of the patients that have CRC present other symptoms than rectal bleeding, change in bowel habits or weight loss when they consult primary care [11]. Furthermore, CRC and inflammatory bowel disease (IBD) can present with the same symptoms [9, 12]. It can be a challenge for primary care doctors to decide which patients to refer to secondary care for further investigation.

Faecal immunochemical tests (FITs) for haemoglobin can be used as triage tests [4, 6, 13–18]. In Sweden, qualitative point of care (POC) dip-stick FITs are in common use in primary care. We have previously shown that the combination of a POC FIT and haemoglobin analysis is a sensitive method for detecting CRC in symptomatic patients in primary care [12]. Therefore, understanding how doctors have interpreted and recorded patients´ symptoms when FITs are requested has importance for the detection of CRC. To our knowledge this has not previously been studied.

The primary aim of the study was to determine the characteristics and extent of patients’ symptoms when FITs are used as triage tests in primary care, and to determine whether doctors recorded the symptoms that patients reported, and the secondary aim was to evaluate in these patients the association between the symptoms, FIT results and possible prediction of CRC or IBD.

## Methods

We conducted a prospective study in the region of Jämtland Härjedalen in Sweden, including four primary care centres covering around 29.000 people. Between 30 January 2013 and 31 May 2014, consecutive patients aged 20 years and older for whom a doctor requested a FIT were invited to take part in the study. Trained nurses informed eligible patients, distributed patients´ information sheets with thorough information about the purpose of the study and ensured that each patient was able to read this (Additional file 1). Questionnaires and tests were distributed to those consenting to participate. The nurses instructed the patients on how to collect the faecal samples. The patients’ consents were verbal, and only patients that returned their questionnaires were included in the study. This procedure, including the written information and the verbal consent, was approved by the Regional Ethical Review Board Umeå. At the time of the study there were no Swedish guidelines regarding suspected CRC, no earlier or on-going screening for CRC in the region and none of the patients had participated in screening programmes. Further details on methods, power calculation and participants have been published previously [12]. The study was performed according to the STARD guidelines [19].

### Questionnaires

#### Patients’ questionnaires

Binary variables (yes/no) were used for questions about alarm symptoms during the previous year. These questions included rectal bleeding in toilet, rectal bleeding on toilet paper, black faeces, change in bowel habits and weight loss. To explore other common gastrointestinal symptoms, we used the validated Gastrointestinal Symptom Rating Scale for irritable bowel syndrome (GSRS-IBS) with thirteen questions, and also five questions from the original GSRS questionnaire [20, 21]. The questions in the GSRS questionnaires use a 7-point Likert scale and ask about symptoms perceived during the previous week. The answers were grouped into seven clusters: abdominal pain, constipation, diarrhoea, reflux symptoms, bloating, satiety and dyspepsia. In each cluster the result of the question with the highest numerical value was recorded. The results from the two questions about incomplete evacuation and urgency were treated separately. The patients’ questionnaire is presented in Additional file 2.

#### Doctors’ questionnaires

After the consultation, the doctor was asked to complete a questionnaire including symptoms and findings that the doctor had noted during the consultation. Four questions corresponded to the patients’ questions about rectal bleeding, black faeces, change in bowel habits and weight loss, six questions corresponded to the symptom clusters and questions of the GSRS-IBS, and two questions to examination findings. The questions could be answered with “yes”, “no” or “unknown/not examined”. The doctors’ questionnaire is presented in Additional file 3.

#### Faecal immunochemical tests (FITs)

The FIT used was the qualitative test Actim Fecal Blood (Oy Medix Biochemica Ab, Finland). This visually interpreted, immunochromatographic dip-stick test was the faecal occult blood test used at all primary care centres as well as the central laboratory at the regional hospital in Jämtland Härjedalen at the time of the study. The laboratories at the primary care centres are all supervised by the Department of Laboratory Medicine at Östersund Hospital (the regional hospital). Each test stick had a built-in control line for quality control of the chromatographic process. The collection tube had a sampling stick attached to the cap which collected an expected mass of 10–20 mg faeces in 10 ml buffer solution [22]. Patients were instructed by experienced laboratory nurses to twist the stick randomly in several different places in the faeces, to store the samples in a refrigerator and to deliver them as soon as possible to the primary care centre. The FITs were analysed on arrival at each primary care centre by experienced laboratory staff, who had no access to clinical information. According to the manufacturer at the time of the study, the cut-off value for a positive result was 50 ng haemoglobin/ml of faecal solution corresponding to 25–50 μg haemoglobin/g faeces and test results remained positive up to 500 ng haemoglobin/ml [22]. The range given of the cut-off value was due to the possible variations in the weight of the collected faeces. Each FIT consisted of three samples from consecutive bowel movements and a result was considered positive when at least one of the samples showed a positive reading. It was in the region customary to request three samples for one FIT.

### Significant colorectal disease

Doctors were instructed to refer patients with positive FITs for a colonoscopy and otherwise to follow their usual procedures. Significant colorectal disease was defined as CRC, adenomas with high-grade dysplasia (HGD), adenomas ≥1 cm with low-grade dysplasia (LGD), or IBD. Such adenomas were recorded as they can be precursors to CRC. The FIT results were available to the endoscopists. All patients were followed for 2 years. Data regarding colonoscopies, sigmoidoscopies, CT colonographies, double contrast barium enemas and diagnoses was retrieved from patients’ electronic medical records that were shared across the primary care centres and all hospital departments. If no CRC or IBD was diagnosed during the two-year follow-up, the patients were considered as not having these diseases.

### Statistics

We used SPSS version 24 for statistical analyses (IBM, Armonk, NY, USA). Comparisons were made using Pearson’s Chi-square test with Yate’s continuity correction or Fisher’s exact test, as appropriate. A *p*-value of < 0.05 was considered significant. As not all patients were investigated with bowel imaging, positive predictive values (PPV) and negative predictive values (NPV) with 95% confidence intervals (CI) for FITs and rectal bleeding were calculated only for the diagnoses of CRC and IBD. The relationship between CRC and IBD, and reported symptoms together with FIT results was explored using Cox regression analysis. The presence of CRC or IBD was used as a dependent variable. Sex and variables with a *p* < 0.10 were included in the analysis as independent dichotomised variables and age was included as a continuous variable. Patient-reported and doctor-reported symptoms were calculated separately. The result of the Cox regression analysis is presented as hazard ratio (HR) with 95% CI. Due to the exploring nature of the study we did not correct for multiple testing.

## Results

**Fig. 1:**
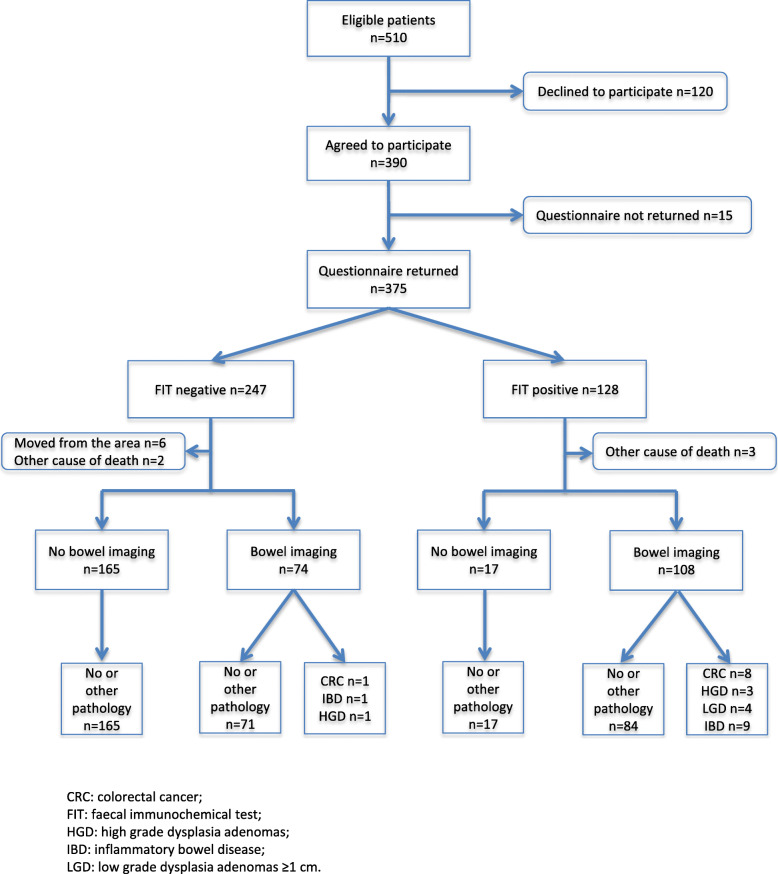
Study flow diagram

Of 510 eligible patients 375 returned the questionnaire. Six of these moved from the region and five died of other causes during the follow-up. 364 patients (64.3% women, median age 64 years) were included in the analysis (Fig. 1). In 356 of these cases, the doctor’s questionnaire was returned. In 135 cases the patient declined to participate or did not return the questionnaire (65.8% women, median age 65 years).

The results of the answers are presented in Table 1. The most common symptom reported by patients was bloating, followed by abdominal pain. Defecation abnormalities were also frequently reported. The symptom most frequently recorded by doctors was abdominal pain, however in general the doctors recorded fewer symptoms than the patients. Almost half of the patients (43.5%) reported rectal bleeding, while this symptom was recorded by 25.6% of the doctors.

**Table 1:** Symptoms and findings reported by patients and doctors respectively, when a faecal immunochemical test (FIT) was requested in primary care

		Patients	Doctors *N* = 356		
Symptoms	N	Mild discomfort or more (%)	Yes (%)	No (%)	Unknown (%)
Abdominal pain^a^	364	266 (73.1)	201 (56.5)	149 (41.9)	6 (1.7)
Diarrhoea^a^	361	212 (58.7)	151 (42.4)	191 (53.7)	14 (3.9)
Urgency^a^	360	186 (51.7)	72 (20.2)	249 (69.9)	35 (9.8)
Constipation^a^	359	189 (52.6)	94 (26.4)	246 (69.1)	16 (4.5)
Incomplete evacuation^a^	361	198 (54.8)	55 (15.4)	253 (71.1)	48 (13.5)
Reflux symptoms^a^	362	122 (33.7)	44 (12.4)	262 (73.6)	50 (14.0)
Bloating^a^	362	303 (83.7)			
Dyspepsia^a^	363	202 (55.6)			
Satiety^a^	361	168 (46.5)			
Change in bowel habits^b^	342	164 (48.0)	158 (44.4)	188 (52.8)	10 (2.8)
Rectal bleeding^b^	347	151 (43.5)	91 (25.6)	245 (68.8)	20 (5.6)
Black faeces^b^	338	72 (21.3)	20 (5.6)	317 (89.0)	19 (5.3)
Weight loss^b^	343	65 (19.0)	45 (12.6)	288 (80.9)	23 (6.5)
Anaemia^ad^			62 (17.4)	227 (63.8)	67 (18.8)
Abdominal mass^a^			4 (1.1)	320 (89.9)	32 (9.0)
One or more alarm symptom^c^	352	260 (73.9)	229 (64.3)		
Two or more alarm symptoms^c^	345	100 (29.0)	58 (16.3)		
Three alarm symptoms^c^	340	15 (4.4)	2 (0.6)		

^a^Questions answered on a scale from “no discomfort” to “very severe discomfort” by patients, and with “yes”, “no” or “unknown” by doctors

^b^Question answered with “yes” or “no” by patients and with “yes”, “no” or “unknown” by doctors

^c^Alarm symptoms: Rectal bleeding, change in bowel habits, weight loss

^d^As known when the FITs were requested

Agreeing positive answers from the patient and the doctor, to the four questions answered with yes/no by the patient and yes/no/unknown by the doctor, varied from 22.9% (black faeces) to 60.9% (change of bowel habits) (Table 2). In general, if a patient rated a symptom as more severe, the doctor more often reported the same symptom. For questions with grading of symptoms and where patients had noted “moderate discomfort” or worse, the highest percentage of agreeing positive answers was for abdominal pain (72,2%).

**Table 2:** Agreeing positive answers from patient and doctor, stratified for grading of symptoms as stated by the patient

		Positive answers for different grading of symptoms by patients											
		Minor discomfort or more		Mild discomfort or more		Moderate discomfort or more		Moderately severe discomfort or more		Severe discomfort or more		Very severe discomfort	
Symptoms	N	Patient	Both (%)	Patient	Both (%)	Patient	Both (%)	Patient	Both (%)	Patient	Both (%)	Patient	Both (%)
Abdominal pain^a^	356	305	194 (63.6)	262	183 (69.8)	216	156 (72.2)	105	88 (83.8)	34	29 (85.3)	6	5 (83.3)
Diarrhoea^a^	353	258	139 (53.9)	208	124 (59.6)	169	110 (65.1)	102	79 (77.5)	52	46 (88.5)	18	17 (94.4)
Urgency^a^	352	227	66 (29.1)	182	63 (34.6)	143	59 (41.3)	99	48 (48.5)	46	24 (52.2)	10	8 (80.0)
Constipation^a^	351	243	86 (35.4)	185	77 (41.6)	145	67 (46.2)	84	51 (60.7)	38	27 (71.1)	15	12 (80.0)
Incomplete evacuation^a^	353	259	52 (20.1)	194	48 (24.7)	160	41 (25.6)	81	22 (27.2)	36	13 (36.1)	10	6 (60.0)
Reflux symptoms^a^	354	175	39 (22.3)	120	26 (21.7)	75	20 (26.7)	36	12 (33.3)	15	6 (40.0)	5	1 (20.0)
Change in bowel habits^b^	335	161	98 (60.9)										
Rectal bleeding^b^	339	148	84 (56.8)										
Black faeces^b^	330	70	16 (22.9)										
Weight loss^b^	335	62	35 (56.5)										

^a^Questions answered on a scale from “no discomfort” to “very severe discomfort” by patients, and with “yes”, “no” or “unknown” by doctors

^b^Question answered with “yes” or “no” by patients and with “yes”, “no” or “unknown” by doctors

All patients included in the study also provided faecal samples for the FITs. When patients recorded rectal bleeding, FITs were negative in 58.3% of cases; when doctors recorded rectal bleeding, FITs were negative in 52.7% (Table 3). Two of the patients that recorded rectal bleeding and had negative FITs had significant colorectal disease: one patient with CRC in the proximal colon recorded blood on the toilet paper, and one with a HGD adenoma recorded blood in the toilet. Both these patients had anaemia according to the hospital laboratory’s definition (haemoglobin < 117 g/l in women, < 134 g/l in men). The PPV of a FIT combined with patient-reported rectal bleeding was 22.6% (95% CI 12.2–33.0) for CRC or IBD. In patients that denied rectal bleeding, the FIT had a PPV of 5.4% for CRC or IBD.

**Table 3:** Patients´ and doctors’ reports of rectal bleeding alone and in combination with FIT results, stratified for diagnoses of colorectal cancer and inflammatory bowel disease

	Rectal bleeding reported	Rectal bleeding reported only			Rectal bleeding reported and FIT						Rectal bleeding denied	Rectal bleeding denied and FIT					
					FIT positive			FIT negative				FIT positive			FIT negative		
	N	No CRC or IBD	CRC + IBD	PPV % (CI)	No CRC or IBD	CRC + IBD	PPV % (CI)	No CRC or IBD	CRC + IBD	NPV % (CI)	N	No CRC or IBD	CRC + IBD	PPV % (CI)	No CRC or IBD	CRC + IBD	NPV % (CI)
Patients	151	136	15	9.9 (5.2–14.7)	48	14	22.6 (12.2–33.0)	88	1	98.9 (96.7–100)	196	53	3	5.4 (0–11.3)	139	1	99.3 (97.9–100)
Blood only on paper	71	65	6	8.5 (2.0–14.9)	19	5	20.8 (4.6–37.1)	46	1	97.9 (93.8–100)	NA	NA	NA	NA	NA	NA	NA
Blood in toilet	80	71	9	11.3 (4.3–18.2)	29	9	23.7 (10.2–37.2)	42	0	100 (98.9–100)	NA	NA	NA	NA	NA	NA	NA
Doctors	91	80	11	12.1 (5.4–18.8)	32	11	25.6 (12.5–38.6)	48	0	100 (99.0–100)	245	66	6	8.3 (2.0–14.7)	173	0	100 (99.4–100)

*CRC* Colorectal cancer

*FIT* Faecal immunochemical test

*IBD* Inflammatory bowel disease

*NPV* Negative predicitive value for CRC + IBD

*PPV* Positive precictive value for CRC + IBD

*CI* 95% confidence interval

*NA* Not applicable

For patient-recorded change in bowel habits the PPV for CRC or IBD was 6.1% (95% CI 2.4–9.8); for change in bowel habits combined with a FIT the PPV was 18.2% (95% CI 9.1–30.9) and the NPV was 100% (95% CI 96.7–100). For patient-recorded weight loss the PPV for CRC or IBD was 10.8% (95% CI 3.2–18.3); for weight loss combined with a FIT the PPV was 24.1% (95% CI 10.3–43.5) and the NPV 100% (90.3–100).

Bowel imaging was performed on 182 patients (Fig. 1). Of the 62 patients that recorded rectal bleeding and had positive FITs, 53 (85.5%) were investigated (50 with colonoscopy and three with CT colonography). Of the 89 patients that recorded rectal bleeding and had negative FITs, 32 (36.0%) were investigated (23 with colonoscopy, one with CT colonography, and eight with a barium enema). In addition, rigid rectoscopy was performed on eleven patients that recorded rectal bleeding (five with positive FITs and six with negative FITs). In total, significant colorectal disease was diagnosed in 27 patients. There were no adverse events during bowel imaging.

Table 4 shows symptoms recorded by patients and doctors stratified for outcome. CRC and IBD were related to rectal bleeding recorded by patients and by doctors.

**Table 4:** Symptoms reported by patients and doctors when a faecal immunochemical test (FIT) was requested in primary care, stratified for outcome

		FIT	Significant colorectal disease				CRC or IBD			
Symptom (n)	With symptom	FIT positive (%)	Sign CRD, total	Symptom + no sign CRD (% of total without CRD)	Symptom + sign CRD (% of total with CRD)	*p*	CRC or IBD, total	Symptom + no CRC or IBD (% of total without CRC/IBD)	Symptom + CRC or IBD (% of total with CRC/IBD)	*p*
Abdominal pain										
Patient (364)	220^a^	74 (33.6)	27	206 (61.1)	14 (51.9)	0.46	19	210 (60.9)	10 (52.6)	0.64
Doctor (350)	201	58 (28.9)	26	187 (57.7)	14 (53.8)	0.86	18	193 (58.1)	8 (44.4)	0.37
Diarrhoea										
Patient (361)	173^a^	63 (36.4)	27	157 (47.0)	16 (59.3)	0.31	19	160 (46.8)	13 (68.4)	0.11
Doctor (342)	151	54 (35.8)	26	134 (42.4)	17 (65.4)	0.04	18	139 (42.9)	12 (66.7)	0.08
Urgency										
Patient (360)	146^a^	56 (38.4)	26	133 (39.8)	13 (50.0)	0.42	19	135 (39.6)	11 (57.9)	0.18
Doctor (321)	72	30 (41.7)	23	64 (21.5)	8 (34.8)	0.23	16	66 (21.6)	6 (37.5)	0.21
Constipation										
Patient (359)	147^a^	52 (35.4)	26	134 (40.2)	13 (50.0)	0.44	19	138 (40.6)	9 (47.4)	0.73
Doctor (340)	94	29 (30.9)	26	89 (28.3)	5 (19.2)	0.44	18	91 (28.3)	3 (16.7)	0.42
Incomplete evacuation										
Patient (361)	162^a^	59 (36.4)	26	149 (44.5)	13 (50.0)	0.73	19	153 (44.7)	9 (47.4)	1.00
Doctor (308)	55	20 (36.4)	22	52 (18.2)	3 (13.6)	0.78	14	55 (18.7)	0	0.08
Reflux symptoms										
Patient (362)	76^a^	21 (27.6)	27	73 (21.8)	3 (11.1)	0.29	19	74 (21.6)	2 (10.5)	0.39
Doctor (306)	44	9 (20.5)	21	40 (14.0)	4 (19.0)	0.52	15	43 (14.8)	1 (6.7)	0.71
Change in bowel habits										
Patient (342)	164	55 (33.5)	26	150 (47.5)	14 (53.8)	0.67	19	154 (47.7)	10 (52.6)	0.85
Doctor (346)	158	60 (38.0)	26	144 (45.0)	14 (53.8)	0.51	18	149 (45.4)	9 (50.0)	0.89
Rectal bleeding										
Patient (347)	151	62 (41.1)	27	132 (41.3)	19 (70.4)	0.006	19	136 (41.5)	15 (78.9)	0.003
Doctor (336)	91	43 (47.3)	24	79 (25.3)	12 (50.0)	0.02	17	80 (25.1)	11 (64.7)	0.001
Black faeces										
Patient (338)	72	30 (41.7)	27	66 (21.2)	6 (22.2)	1.00	19	68 (21.3)	4 (21.1)	1.00
Doctor (337)	20	11 (55.0)	26	17 (5.5)	3 (11.5)	0.19	18	19 (6.0)	1 (5.6)	1.00
Weight loss										
Patient (343)	65	29 (44.6)	27	57 (18.0)	8 (29.6)	0.22	19	58 (17.9)	7 (36.8)	0.06
Doctor (333)	45	20 (44.4)	24	42 (13.6)	3 (12.5)	1.00	16	43 (13.6)	2 (12.5)	1.00

^a^Moderate discomfort or more

*P* value was calculated with Pearson’s Chi-square test or Fisher’s exact test

Significant colorectal disease: Colorectal cancer, adenomas with high-grade dysplasia, adenomas with low-grade dysplasia ≥1 cm, inflammatory bowel disease

*CRC* colorectal cancer. *IBD* inflammatory bowel disease. *CRD* colorectal disease

Using Cox regression analysis, we related reported symptoms and the FIT results to the diagnoses of CRC/IBD (dependent factor). In the Cox regression analysis of patient-reported symptoms a positive FIT (HR 15.8; CI 3.65–69.2), female sex (HR 3.28; 95% CI 1.05–10.3), reported rectal bleeding (HR 7.47; CI 2.16–25.8), reported diarrhoea (HR 2.78; CI 1.03–7.52) and reported weight loss (HR 3.68; CI 1.35–10.1) was significantly associated to CRC/IBD. In the analysis of doctor-reported symptoms only a positive FIT (HR 15.8; CI 3.59–70.1) and reported rectal bleeding (HR 3.03; CI 1.06–8.65) was associated to CRC/IBD.

## Discussion

Abdominal pain was the most common symptom reported by patients as well as by doctors. Diarrhoea and change in bowel habits were also frequently reported. Rectal bleeding was recorded by 43.5% of the patients and 25.6% of the doctors. The concordance between patient-recorded and doctor-recorded symptoms was generally low, with the highest percentage of agreeing answers for abdominal pain. The combination of a doctor’s positive history of patient rectal bleeding in combination with a positive FIT showed a high PPV of 25.6% (95% CI 12.5–38.6) for CRC and IBD. Rectal bleeding was the only symptom reported by both patients and doctors that was associated to CRC/IBD.

Unexpectedly, almost half of the patients recorded rectal bleeding. To our knowledge, only one previous study has reported on patients’ symptoms (doctor-reported) when FITs were requested in primary care [23]. In this Danish study, that presents no information about rectal bleeding and that did not include patients eligible for urgent referral, the most frequent symptoms were abdominal pain (53.9%) and change in bowel habits (45.6%). These figures are in line with the present study.

Not surprisingly, the percentage of agreeing positive answers from patients and doctors increased with the severity of symptoms recorded by patients. However, even when “severe discomfort” or worse was recorded by patients, in 15% of cases doctors had not recorded abdominal pain, and in 11% of cases they had not recorded diarrhoea. A low concordance between patient-reported and doctor-recorded symptoms has also been seen in studies concerning other conditions [24, 25]. The percentage of agreeing positive answers to questions about urgency and incomplete evacuation of faeces was low, and a higher proportion of the doctors answered these questions with “unknown”. It is probable that doctors considered these symptoms to be less important.

There was a substantial discrepancy between numbers of patients and doctors that recorded rectal bleeding, and the percentage of agreeing positive answers was as low as 56.8%. This may have several explanations: other symptoms may have dominated, the bleeding may have been interpreted as caused by unimportant haemorrhoids, patients may be less willing to talk about rectal issues than other symptoms, and doctors may have forgotten to ask about bleeding. A former study with patients diagnosed with rectal cancer reported that over 50% initially attributed their symptoms to haemorrhoids and thought that the symptoms were not serious [26]. This indicates that patients may hesitate to mention rectal bleeding in the consultation.

In spite of the difference in recording of rectal bleeding, this was the only symptom that was significantly associated with the diagnoses of CRC or IBD, both when recorded by patients and by doctors. This relationship has also been found in previous studies [27]. As it seems important not to miss rectal bleeding, further research on how to improve communication between patients and doctors on this subject could be useful.

However, over 50% of the cases with recorded rectal bleeding had negative FITs, both when the bleeding was recorded by the doctor and by the patient, and only one patient that recorded rectal bleeding and had a negative FIT had CRC. (In this case it was also unlikely that the bleeding was caused by the CRC, as the patient reported blood on the toilet paper and had a right-sided tumour.) Adding FIT results to the history of rectal bleeding increased the PPV for CRC and IBD substantially and also gave a high NPV. Previous studies have shown that FITs may be useful in prioritising patients that had already been referred to secondary care [28–34]. A recently published study shows similar results when FITs were requested in primary care [35]. Guidelines on suspected CRC instruct primary care doctors to refer patients with unexplained rectal bleeding. It can be challenging for primary care doctors to rule out CRC in patients with rectal bleeding and findings of haemorrhoids. In these cases, a negative FIT combined with an anorectal investigation could help rule out CRC in a primary care setting. Interestingly, FITs have already been used in these situations by doctors as a triage tests in primary care in Sweden [36].

Irrespective of blood being recorded to occur on the paper only or in the toilet, the PPVs and NPVs for CRC and IBD were similar – thus it seems to be of less importance to distinguish between different locations of blood.

Reports of a change in bowel habits, as an isolated symptom, was not associated with significant colorectal disease in this study. Instead diarrhoea may be more connected to significant bowel disease. Constipation was not associated with significant bowel disease, which is in line with earlier findings [37].

Our study has several limitations. Firstly, the study starts from the consultation when the doctor requested a FIT and we cannot rule out that some patients prior to the inclusion had consulted a doctor for gastrointestinal symptoms. Also, patients may have been referred for endoscopy without previous testing. However, the patients included are those where doctors presumably were in need of a diagnostic aid. Secondly, patients were asked to record symptoms during a specified period, while doctors were asked to record symptoms presented at the consultation. This may to some extent have effected the percentage of agreeing positive answers for patient-reported and doctor-reported symptoms in our study. Thirdly, not all patients underwent bowel imaging. However, due to the two-year observation period it is unlikely that any cases of CRC or IBD were missed. Also, of the eligible patients, 26% did not consent to participate or return the questionnaire and possibly the non-participants could have differed from those in the participating group. However, age and sex distribution were similar in the two groups.

The FIT used in this study was qualitative with a cut-off of 25–50 μg haemoglobin/g (Hb/g) faeces. Previous studies on already referred patients have shown that a qualitative POC FIT with a cut-off of < 6 μg Hb/g and quantitative FITs with cut-offs from detectable blood to 15 μg Hb/g faeces could with reasonable safety rule out significant colorectal disease [28, 30, 32, 33]. Thus, considerably lower cut-off values were used in these studies. In England, the NICE diagnostic guidance with recommendations on referral for suspected CRC in primary care recommends quantitative FITs with cut-offs of 10 μg Hb/g for patients with low risk symptoms [13]. A Danish study on patients where FITs were requested by doctors before referral used a quantitative FIT with a cut-off of 10 μg Hb/g faeces and showed a PPV of 11.5% for CRC and IBD in patients with non-alarm symptoms [23]. As the clinical observation time after a negative FIT was only 3 months the NPV in that study seems uncertain. For the use of FITs as rule-out tests in primary care, it is important to find the optimal cut-off level so as to prevent unnecessary referrals and avoid missing cases of significant colorectal disease, especially CRC. Further studies are necessary to determine this level. The combination of a FIT with a haemoglobin value could be useful [12].

## Conclusion

Doctors should be aware that, during consultations, they do not record all symptoms experienced by patients. FITs requested by doctors in primary care when found positive may potentially be of help in prioritising referrals, also when patients present with the alarm symptoms of rectal bleeding or change in bowel habits.

## Supplementary information

**Additional file 1.** Patients information

**Additional file 2.** Questions about gastrointestinal symptoms

**Additional file 3.** Questions to the doctor who has requested a Faecal Immunochemical Test (FIT) for this patient

## Data Availability

The datasets used and analysed during the current study are available from the corresponding author on reasonable request.

## Abbreviations

CRC: Colorectal cancer
CRD: Colorectal disease
FIT: Faecal immunochemical test
GSRS-IBS: Gastrointestinal Symptom Rating Scale for irritable bowel syndrome
HGD: High-grade dysplasia
IBD: Inflammatory bowel disease
LGD: Low-grade dysplasia
OR: Odds ratio
NPV: Negative predictive value
POC: Point of care
PPV: Positive predictive value
