# Supplementary material for: Patient-reported and doctor-reported symptoms when faecal immunochemical tests are requested in primary care in the diagnosis of colorectal cancer and inflammatory bowel disease: a prospective study
Source: BMC Fam Pract. 2020 Jul 1;21:129. doi: 10.1186/s12875-020-01194-x (PMC7331274; doi:10.1186/s12875-020-01194-x)
Supplement: Supplementary file 1 — Additional file 1. Patients information [file 12875_2020_1194_MOESM1_ESM.docx]

# **Patients’ information** (translated from Swedish)

Abdominal and bowel symptoms are common and usually have no serious background. It is none the less important to find the persons with illnesses that should be treated and where further investigations are needed. Tests for blood in faeces (stools) are commonly used as an aid in decision on further investigation. Red blood cell counts may also contribute. However, the test results can be difficult to evaluate.

Four health centres in Jämtland are now taking part in a research project connected to Umeå University with the aim to investigate how the testing may be improved. We also examine if certain combinations of symptoms and test results could be connected to an increased risk of disease.

We now ask if you would like to take part in this research project. Your doctor has already requested you to collect stool samples to examine if they contain blood. If you take part in the project you are requested to also collect a stool sample for a test called F-Calprotectin, which can detect bowel inflammation. We will also take blood samples for a full blood count and an iron test.

We also ask you to complete a questionnaire about abdominal and bowel symptoms, and what medications you currently use (some medicines may have influence on the test results).

Your participation in the study is completely voluntary, and your answers and test results will be treated so that no unauthorised person can get access to it. The test results will be registered in your patient record as usual. There will be no information about personal identity in data sets, data processing will be coded. All data will be treated in accordance with the Swedish Personal Data Protection Act, the County Council of Jämtland has responsibility for this. You can at any time get information about what is registered about you (register transcript) and have data corrected. Contact person for personal data is Cecilia Högberg, for contact details please see below.

The study is approved by the Regional Ethical Review Board in Umeå. The research results will be reported to the County Council of Jämtland and presented internationally.

You are very welcome to contact one of us if you have any questions or want to know more about the research project.

Please return the questionnaire together with the stool samples to the health centre. Those who have not returned stool samples and questionnaire within two weeks will receive one reminder.

Thank you,

Cecilia Högberg Mikael Lilja

Specialist Family Medicine Specialist Family Medicine

Krokom Health Centre Unit of Research and Education

tel 0640-16600 Jämtland County Council
