# Supplementary material for: Patient-reported and doctor-reported symptoms when faecal immunochemical tests are requested in primary care in the diagnosis of colorectal cancer and inflammatory bowel disease: a prospective study
Source: BMC Fam Pract. 2020 Jul 1;21:129. doi: 10.1186/s12875-020-01194-x (PMC7331274; doi:10.1186/s12875-020-01194-x)
Supplement: Supplementary file 2 — Additional file 2. Questions about gastrointestinal symptoms [file 12875_2020_1194_MOESM2_ESM.docx]

Date ………. Name ……………... ID-number…….... (translated from Swedish)

**Questions about gastrointestinal symptoms (18 questions)**

| Please read this first:  This questionnaire contains questions about how you have been feeling and what it has been like during the past week. Mark the choice that best  applies to you and your situation with an X. |
| --- |

1. Have you been bothered by ABDOMINAL PAIN during the past week?

| No discomfort at all |  |
| --- | --- |
| Minor discomfort |  |
| Mild discomfort |  |
| Moderate discomfort |  |
| Moderately severe discomfort |  |
| Severe discomfort |  |
| Very severe discomfort |  |

2. Have you been bothered by PAIN OR DISCOMFORT IN YOUR ABDOMEN RELIEVED BY A BOWEL ACTION during the past week?

| No discomfort at all |  |
| --- | --- |
| Minor discomfort |  |
| Mild discomfort |  |
| Moderate discomfort |  |
| Moderately severe discomfort |  |
| Severe discomfort |  |
| Very severe discomfort |  |

3. Have you been bothered by A FEELING OF BLOATING during the past week?

| No discomfort at all |  |
| --- | --- |
| Minor discomfort |  |
| Mild discomfort |  |
| Moderate discomfort |  |
| Moderately severe discomfort |  |
| Severe discomfort |  |
| Very severe discomfort |  |

4. Have you been bothered by PASSING GAS during the past week?

| No discomfort at all |  |
| --- | --- |
| Minor discomfort |  |
| Mild discomfort |  |
| Moderate discomfort |  |
| Moderately severe discomfort |  |
| Severe discomfort |  |
| Very severe discomfort |  |

5. Have you been bothered by CONSTIPATION OR PROBLEMS EMPTYING THE BOWEL during the past week?

| No discomfort at all |  |
| --- | --- |
| Minor discomfort |  |
| Mild discomfort |  |
| Moderate discomfort |  |
| Moderately severe discomfort |  |
| Severe discomfort |  |
| Very severe discomfort |  |

6. Have you been bothered by FREQUENT BOWEL MOVEMENTS during the past week?

| No discomfort at all |  |
| --- | --- |
| Minor discomfort |  |
| Mild discomfort |  |
| Moderate discomfort |  |
| Moderately severe discomfort |  |
| Severe discomfort |  |
| Very severe discomfort |  |

7. Have you been bothered by LOOSE BOWEL MOVEMENTS during the past week?

| No discomfort at all |  |
| --- | --- |
| Minor discomfort |  |
| Mild discomfort |  |
| Moderate discomfort |  |
| Moderately severe discomfort |  |
| Severe discomfort |  |
| Very severe discomfort |  |

8. Have you been bothered by HARD STOOLS during the past week?

| No discomfort at all |  |
| --- | --- |
| Minor discomfort |  |
| Mild discomfort |  |
| Moderate discomfort |  |
| Moderately severe discomfort |  |
| Severe discomfort |  |
| Very severe discomfort |  |

9. Have you been bothered by an URGENT NEED TO HAVE A BOWEL MOVEMENT (need to go to the toilet urgently) during the past week?

| No discomfort at all |  |
| --- | --- |
| Minor discomfort |  |
| Mild discomfort |  |
| Moderate discomfort |  |
| Moderately severe discomfort |  |
| Severe discomfort |  |
| Very severe discomfort |  |

10. Have you been bothered by A FEELING THAT YOUR BOWEL WAS NOT COMPLETELY EMPTIED after having a bowel movement during the past week?

| No discomfort at all |  |
| --- | --- |
| Minor discomfort |  |
| Mild discomfort |  |
| Moderate discomfort |  |
| Moderately severe discomfort |  |
| Severe discomfort |  |
| Very severe discomfort |  |

11. Have you been bothered by FEELING FULL SHORTLY AFTER YOU HAVE STARTED A MEAL during the past week?

| No discomfort at all |  |
| --- | --- |
| Minor discomfort |  |
| Mild discomfort |  |
| Moderate discomfort |  |
| Moderately severe discomfort |  |
| Severe discomfort |  |
| Very severe discomfort |  |

12. Have you been bothered by FEELING FULL EVEN LONG AFTER YOU HAVE STOPPED EATING during the past week?

| No discomfort at all |  |
| --- | --- |
| Minor discomfort |  |
| Mild discomfort |  |
| Moderate discomfort |  |
| Moderately severe discomfort |  |
| Severe discomfort |  |
| Very severe discomfort |  |

13. Have you been bothered by VISIBLE SWELLING OF YOUR ABDOMEN during the past week?

| No discomfort at all |  |
| --- | --- |
| Minor discomfort |  |
| Mild discomfort |  |
| Moderate discomfort |  |
| Moderately severe discomfort |  |
| Severe discomfort |  |
| Very severe discomfort |  |

14. Have you been bothered by HEARTBURN during the past week?

| No discomfort at all |  |
| --- | --- |
| Minor discomfort |  |
| Mild discomfort |  |
| Moderate discomfort |  |
| Moderately severe discomfort |  |
| Severe discomfort |  |
| Very severe discomfort |  |

15. Have you been bothered by ACID REFLUX during the past week?

| No discomfort at all |  |
| --- | --- |
| Minor discomfort |  |
| Mild discomfort |  |
| Moderate discomfort |  |
| Moderately severe discomfort |  |
| Severe discomfort |  |
| Very severe discomfort |  |

16. Have you been bothered by DISCOMFORT IN YOUR UPPER

ABDOMEN during the past week?

| No discomfort at all |  |
| --- | --- |
| Minor discomfort |  |
| Mild discomfort |  |
| Moderate discomfort |  |
| Moderately severe discomfort |  |
| Severe discomfort |  |
| Very severe discomfort |  |

17. Have you been bothered by NAUSEA OR VOMITS during the past week?

| No discomfort at all |  |
| --- | --- |
| Minor discomfort |  |
| Mild discomfort |  |
| Moderate discomfort |  |
| Moderately severe discomfort |  |
| Severe discomfort |  |
| Very severe discomfort |  |

18. Have you been bothered by BURPING during the past week?

| No discomfort at all |  |
| --- | --- |
| Minor discomfort |  |
| Mild discomfort |  |
| Moderate discomfort |  |
| Moderately severe discomfort |  |
| Severe discomfort |  |
| Very severe discomfort |  |

Have you been bothered by any of these symptoms during the past year?

|  | Yes | No |
| --- | --- | --- |
| Blood on the toilet paper after defecation? |  |  |
| Visible blood or blood mixed with stools when you look in the toilet? |  |  |
| Black stools? |  |  |
| Change in bowel habits? |  |  |
| Unintentional weight loss? |  |  |

Do you currently use any of these medications?

Anticoagulants or antiplatelet medicines (for example Waran, Trombyl, Persantin, Clopidogrel, Heparin)?

If yes, which?........................................................................................................................

…………………………………………………………………………………………………...

Painkillers or anti-inflammatory (NSAID) drugs (including over-the-counter drugs)?

If yes, which?........................................................................................................................

…………………………………………………………………………………………………...

Medication for high blood pressure or heart problems?

If yes, which?........................................................................................................................

…………………………………………………………………………………………………...

**Thank you!**
