# Supplementary material for: Patient-reported and doctor-reported symptoms when faecal immunochemical tests are requested in primary care in the diagnosis of colorectal cancer and inflammatory bowel disease: a prospective study
Source: BMC Fam Pract. 2020 Jul 1;21:129. doi: 10.1186/s12875-020-01194-x (PMC7331274; doi:10.1186/s12875-020-01194-x)
Supplement: Supplementary file 3 — Additional file 3. Questions to the doctor who has requested a Faecal Immunochemical Test (FIT) for this patient [file 12875_2020_1194_MOESM3_ESM.docx]

# Patient:……………………………………..(ID) (Translated from Swedish)

# Questions to the doctor who has requested a Faecal Immunochemical Test (FIT) for this patient

Four health centres in Jämtland Härjedalen in cooperation with Umeå University are taking part in a study with the aim of investigating the value of patients’ histories and FIT results for the diagnosis of colorectal cancer, and if a test for faecal calprotectin can improve the diagnosis. The study has been approved by the Regional Ethical Review Board Umeå, and all answers are treated anonymously. I hope you can answer the following questions about this patient for whom you recently requested a FIT.

Did the patient have any of the following symptoms or findings?

**Yes No Unknown/**

**not examined**

1. Abdominal pain? □ □ □

2. Hard faeces? □ □ □

3. Loose bowel movements? □ □ □

4. Urgent need to have a bowel movement

(need to go to the toilet urgently)? □ □ □

5. Feeling that the bowel was not completely emptied? □ □ □

6. Change in bowel habits? □ □ □

7. Heartburn or acid reflux? □ □ □

8. Blood on the toilet paper or in the toilet? □ □ □

9. Black faeces? □ □ □

10. Unintentional weight loss? □ □ □

11. Abdominal tumour? □ □ □

12. Anaemia? □ □ □

13. Other symptom or finding that caused the request of a FIT?

…………………………………………………………………………………..

# Thank you for your participation.

Cecilia Högberg, Specialist family medicine, Hälsocentralen Krokom. Tel 0640-16600.
